# Chromosomal Abnormalities in Myelodysplastic Syndrome Patients in Upper Northern Thailand

**DOI:** 10.31557/APJCP.2020.21.3.639

**Published:** 2020-03

**Authors:** Suphitcha Phrommin, Adisak Tantiworawit, Thanawat Rattanathammethee, Chaniporn Puaninta, Nathaporn Pangjaidee, Sirinda Aungsuchawan, Kanokkan Bumroongkit

**Affiliations:** 1 *Department of Anatomy, *; 2 *Division of Hematology, Department of Internal Medicine, Faculty of Medicine, Chiang Mai University, Chiang Mai, Thailand. *

**Keywords:** Myelodysplastic syndrome, conventional cytogenetic technique, chromosomal abnormalities

## Abstract

**Objective:**

Chromosome detection is important in the diagnosis and prognosis of Myelodysplastic syndrome (MDS) patients. About 50% of MDS patients have chromosomal abnormalities. Moreover, chromosome 5 and 7 are common genetic abnormalities in MDS patients and use to identify prognosis risk group and the proper treatment in MDS patients. The objective of this study was to evaluate chromosomal abnormalities and clinical features of MDS patients in upper northern Thailand.

**Methods:**

Fifty bone marrow (BM) specimens were examined by conventional cytogenetic (CC) technique and fluorescence in situ hybridization (FISH) technique for detected chromosome 5 and 7 abnormalities. The clinical features were comparison between MDS patients with chromosomal abnormalities and those with normal karyotype.

**Results:**

Chromosomal abnormalities were detected in 8/50 MDS patients by CC and 17/50 cases by FISH technique. When the CC and FISH techniques were combined, chromosomal abnormalities increased to 21/50 cases. Abnormalities of isolated chromosome 5 were found in 13 cases and were associated with lower level of percentage blast of BM (*p* = 0.003) and higher level of hemoglobin (*p* = 0.019). Moreover, abnormalities of chromosome 7 were found in 3 cases, 1 case of isolated del(7q) and 2 cases of -7 and del(7q) with complex abnormalities. These three cases were associated with higher level of percentage blast of BM (*p* = 0.010).

**Conclusion:**

This study showed the frequency and pattern of chromosomal abnormalities of MDS patients in upper northern Thailand were different from other populations. MDS with isolated chromosome 5 abnormalities had clinical characteristics corresponding with patients in good prognosis risk group. However, MDS patients with chromosome 7 and complex abnormalities showed higher percentage blast of BM which high risk to progression to acute myeloid leukemia (AML). Combined CC and FISH techniques detect chromosomal abnormalities with greater frequency than when either technique is used alone.

## Introduction

Myelodysplastic syndrome (MDS) are clonal malignancy disorders characterized by ineffective hematopoiesis, dysplastic changes in bone marrow, and peripheral blood cytopenia, and high risk for progression to acute myeloid leukemia (AML) (Delforge, 2003). The classification for diagnosis of MDS patients is based on the World Health Organization (WHO) 2016 by percentage of bone marrow (BM) and peripheral blood (PB) blast cells, dysplastic lineages, peripheral cytopenia, presence of ringed sideroblasts, and cytogenetics by conventional cytogenetic (CC) (Delforge, 2003; Arber et al., 2016). In 2012, the Revised International Prognostic Scoring System (IPSS-R) was used for classifying prognosis of MDS patients. One of the major prognostic criteria is the karyotype of the patient that can be classified patients as very good, good, intermediate, poor, and very poor prognostic risk groups (Greenberg et al., 2012). Previous studies found that about 50% of MDS patients have cytogenetic abnormalities in BM (Solé et al., 2005; Vundinti et al., 2009). Deletion of chromosome 5 and 7 including monosomy/deletion 5q (-5/del(5q)) and monosomy/deletion 7q (-7/del(7q)) are common genetic abnormalities in MDS (Solé et al., 2005; Makishima et al., 2010). The European Cytogenetics Association (E.C.A) recommends detecting -5/del(5q) and -7/del(7q) by fluorescence in situ hybridization (FISH) technique in MDS patients, when chromosome analysis by CC are less than 10 normal metaphase chromosomes (Hastings et al., 2013). The prognosis in MDS patients with del(5q) is classified as a good prognosis whereas MDS with -5 and del(7q) are classified as an intermediate cytogenetic risk group. While, in patients with -7, it is classified as a poor prognosis (Greenberg et al., 2012). Detection of chromosome 5 and 7 abnormalities are assisting the clinician chooses suitable treatment. A specific treatment using lenalidomide is available for MDS patients with del (5q) however patients with chromosome 7 abnormalities require more aggressive treatment (Adema et al., 2013; Komrokji et al., 2013). CC technique is the gold standard technique for detecting all abnormalities of the chromosome, nevertheless it has limitations. This technique requires fresh specimens and cannot detect microscopic deletion or cryptic rearrangement (Bridge, 2008). The FISH technique is a molecular cytogenetic technique to detect specific chromosomal abnormalities and enable study in interphase nucleus (Bridge, 2008; Costa et al., 2010). There are few studies of cytogenetic and clinical features of MDS patients in upper northern Thailand. Moreover, chromosome 5 and chromosome 7 are important for prognosis and therapeutic treatment. Therefore, the objectives of this study were to investigate chromosomal abnormalities by CC technique, and to observe deletion of chromosome 5 and chromosome 7 by FISH technique. Additionally, the clinical features between MDS patients with chromosomal abnormalities and those with normal karyotype were compared.

## Materials and Methods

*Patients and samples*

Fifty bone marrow specimens of adult MDS patients were collected from Division of Hematology, Maharaj Nakhon Chiang Mai Hospital, Thailand. Diagnosis of MDS patients according to WHO 2016 and prognostic risk groups according to IPSS-R were confirmed by hematologist and pathologist. All of specimens were examined for chromosomal abnormalities by CC and FISH techniques. This study was approved by the Ethics and Research Committee of the Faculty of Medicine, Chiang Mai University.

*Conventional cytogenetics technique*

The BM specimens were cultured for 24 hours in RPMI 1640 supplemented with 20% fetal bovine serum, deoxythymidine, antibiotics, and colchicine at 37^o^C. For 30 min before harvest, the cultured specimens were treated with colchicine to stop cell mitosis. After that, chromosomes were harvested, and the metaphase chromosomes were stained with the G-banding or Q-banding technique. Twenty metaphase chromosomes of each patient were analyzed under light microscope for G-band or fluorescence microscope for Q-band. The karyotype was described according to the International System for Chromosome Nomenclature (ISCN 2016). Structure rearrangement or additional chromosome was reported when the same aberration has at least two cells. Deletion of chromosome was reported when the same loss has at least three cells (McGowan-Jordan et al., 2016). 

*Fluorescence in situ hybridization*

FISH was performed on cell suspension from CC technique and using standardized protocols, according to the manufacturer’s instructions. Chromosome 5 abnormalities were detected by Vysis LSI EGR1/D5S23, D5S721 Dual Color Probe Set, targeting at 5q31 and 5p15.2 which labeled in spectrum orange and spectrum green, respectively (Abbott Molecular Inc., Des Plaines, IL, USA). Chromosome 7 abnormalities were detected by Vysis D7S486/CEP 7 FISH Probe Kit targeting at 7q31 and 7p11.1-q11.1 which were labeled in spectrum orange and spectrum green, respectively (Abbott Molecular Inc., Des Plaines, IL, USA). The 200 interphase nuclei of each patient were examined under fluorescence microscope for each probe. FISH signals for the normal number of chromosome 5 or 7 showed two green and two orange signals (2G2R), whereas, a one copy deletion of the long arm of chromosome 5 or 7 showed two green and one orange signals (2G1R), two copies deletion of the long arm of chromosome 5 or 7 showed only 2 green signals (2G0R), and monosomy of chromosome 5 or 7 showed one green and one orange signals in the interphase nuclei (1G1R). The positive results were above the cut-off values which were established for each probe at mean plus 3 standard deviation of 10 normal BM karyotypes. The cut-off values of chromosome 5 for 2G1R, and 1G1R were 5.18%, and 1.34%, respectively. However, the 2G0R signal was not detected in 10 normal BM karyotypes, therefore the cut-off value of this signal was 0.00% and patients who showed this signal were recorded as positive results. The cut-off values of chromosome 7 were 3.95%, 0.52%, and 2.38%, for 2G1R, 2G0R, and 1G1R, respectively.

*Clinical data*

The clinical data of MDS patients such as sex, age, percentage blast of BM, white blood cell (WBC) count, hemoglobin (Hb), platelet count, and absolute neutrophil count (ANC) were obtained from electronic medical record of Maharaj Nakorn Chiang Mai Hospital.

*Statistical analysis*

The frequencies of chromosomal abnormalities were calculated by percentage. The differences of the clinical data were analyzed by Fisher’s exact or Chi-Square test for categorical variables parameters and Wilcoxon-Mann-Whitney U test or Student *t*-test for numerical variables. A *p*-value < 0.05 was considered statistically significant.

## Results

*General data and characteristics*

A total of 50 MDS patients, 28 (56%) were male and 22 (44%) were female. The median age of patients was 66 years (range 28-93 years). The clinical and laboratory features consisting of percentage blast of BM, Hb, WBC count, platelet count, and ANC are listed in Table 1.

*Cytogenetic and FISH results*

**Figure 1:**
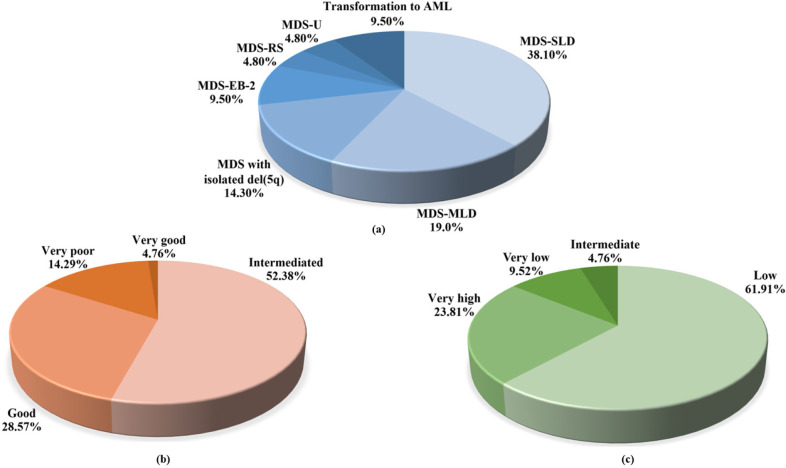
(a) MDS subtypes, (b) cytogenetic risk groups, and (c) IPSS-R risk groups of MDS patients with chromosomal abnormalities

Fifty MDS patients were studied with CC and FISH techniques. Chromosomal abnormalities were detected in 8 of 50 MDS patients (16.0%) by the CC technique. From the FISH technique, the FISH positive signals for chromosome 5 and 7 abnormalities were found in 17 of 50 cases (34.0%). When the CC and FISH techniques were combined, chromosomal abnormalities increased to 21 of 50 cases (42.0%) of patients. From these 21 cases, the chromosomal abnormalities including 1 case of -Y, 1 case of t(2;11)(p21;q23), 1 case of del(20q)(q11.2), 1 case of +8 and +21, 8 cases of isolated -5, 3 cases of isolated del(5q), 2 cases of -5 and del(5q), 1 case of del(5q) with complex abnormalities, 1 cases of isolated del(7q), and 2 cases of chromosome 5 and 7 abnormalities with complex karyotype (Table 2). Patients with chromosomal abnormalities were 8 MDS-SLD (38.10%), 4 MDS-MLD (19.0%), 3 MDS with isolated del(5q) (14.30%), 2 MDS-EB-2 (9.50%),1 MDS-RS (4.80%), 1 MDS-U (4.80%) and 2 transformation to AML (9.50%) (Figure 1a). The prognosis in all these 21 MDS patients were classified by IPSS-R which was based on clinical features and cytogenetic risk group. Cytogenetic risk group of patients with chromosomal abnormalities were classified into 11 intermediated (52.38%), 6 good (28.57%), 3 very poor (14.29%), and 1 very good (4.76%) categories (Figure 1b). When classified by IPSS-R, abnormalities of cytogenetic features consist of 13 low (61.91%), 5 very high (23.81%), 2 very low (9.52%), and 1 intermediate (4.76%) risk groups (Figure 1c). The characteristics of MDS patients with chromosomal abnormalities are summarized in Table 2.

The results of the clinical and laboratory features of MDS patients with normal and abnormal karyotype showed significant difference in gender (*p* = 0.044) and no significant difference in age, percentage blast of BM, Hb, WBC, platelet, and ANC (Table 3).

*Clinical features of patients with chromosome 5 or 7 abnormalities*

When evaluated the clinical and laboratory features of MDS patients with normal chromosome and MDS patients with isolated chromosome 5 abnormalities, which excluded abnormalities of other chromosomes. The result showed significant difference in gender (*p* = 0.041), lower percentage blast of BM (*p* = 0.003) and higher hemoglobin level (*p* = 0.019) than those in normal karyotype (Table 3). Whereas, the clinical and laboratory features of MDS patients with isolated chromosome 7 abnormalities and chromosome 7 with complex karyotype had higher percentage blast of BM (*p* = 0.010) than those MDS patients with normal karyotype by CC and FISH (Table 3).

**Table 1:** General Characteristics of MDS Patients

Characteristics	All patients (n=50)
Gender	
Male, n (%)	28 (56.0)
Female, n (%)	22 (44.0)
Age, median (range)	66 (28-93)
WHO subtype, n (%)	
MDS-SLD	13 (26.0)
MDS-MLD	17 (34.0)
MDS-RS	2 (4.0)
MDS-EB-1	1 (2.0)
MDS-EB-2	8 (16.0)
MDS with isolated del (5q)	3 (6.0)
MDS-U	4 (8.0)
Transformation to AML	2 (4.0)
Clinical data, median (range)	
Percentage blast of BM	1.4 (0.0-90.0)
Hb (g/dL)	8.4 (4.2-13.4)
WBC count (10^9^/L)	4.5 (1.1-209.3)
Platelet count (10^9^/L)	66.5 (2.0-442.0)
ANC (10^9^/L)	2.1 (0.1-30.5)
Cytogenetic categories, n (%)	
Very good	1 (2.0)
Good	35 (70.0)
Intermediate	11 (22.0)
Poor	0 (0.0)
Very poor	3 (6.0)
IPSS-R categories, n (%)	
Very low	3 (6.0)
Low	25 (50.0)
Intermediate	10 (20.0)
High	5 (10.0)
Very high	7 (14.0)

WHO, World Health Organization; MDS-SLD, Myelodysplastic syndrome with single lineage dysplasia; MDS-MLD, Myelodysplastic syndrome with multilineage dysplasia; MDS-RS, Myelodysplastic syndrome with ring sideroblasts; MDS-EB-1, Myelodysplastic syndrome with excess blasts - 1; MDS-EB-2, Myelodysplastic syndrome with excess blasts - 2; MDS with isolated del(5q), Myelodysplastic syndrome with isolated del(5q); MDS-U, Myelodysplastic syndrome unclassifiable; Transformation to AML, transformation to acute myeloid leukemia; Percentage blast of BM, percentage blasts of bone marrow; Hb, Hemoglobin; WBC, white blood cells; ANC, absolute neutrophil count; IPSS-R, Revised International Prognostic Scoring System.

**Figure 2:**
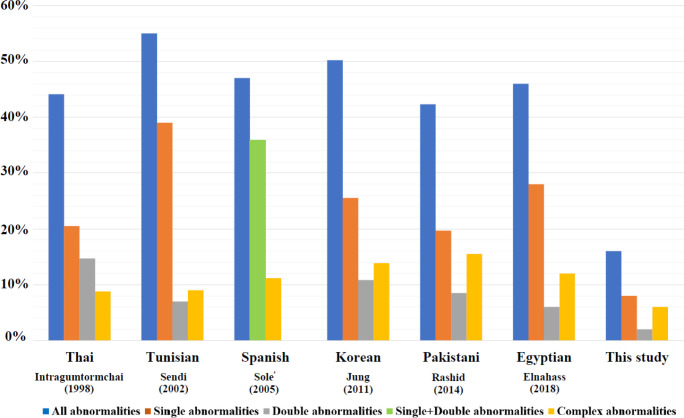
Chromosomal Abnormalities in Each Population by CC Technique

**Table 2:** Cytogenetic Features of 21 MDS Patients from CC and FISH

No	Gender /Age	WHO	Karyotype by CC	FISH signal				Cytogenetic	IPSS-R
		subgroup		-5	del(5q)	-7	del(7q)	categories	
2	M/63	MDS-MLD	45,X,-Y[19]/46,XY[1]	N	N	N	N	Very good	Very low
7	M/78	MDS with isolated del(5q)	46,XY[20]	N	P	N	N	Good	Low
8	F/61	MDS-SLD	46,XX[20]	P	N	N	N	Intermediate	Low
11	F/70	MDS-RS	42 ~48,XX,der(5)t(5;?)(q13;?),-7,+8,der(10) t(10;?)(q22;?),del(12)(q23),der(13)t(13;?) (p11.2;?),der(15)t(15;?)(p11.2;?),-18,-19,-20, -20,+mar1,+mar2,+mar2×2,+mar3[cp19]/ 46,XY[1]	P	P	P	P	Very poor	Very high
12	F/77	MDS-MLD	46,XX[20]	P	N	N	N	Intermediate	Low
14	M/48	MDS-SLD	46,XY,t(2;11)(p21;q23)[20]	N	N	N	N	Intermediate	Low
15	F/57	MDS with isolated del(5q)	46,XX[20]	N	P	N	N	Good	Low
16	F/73	MDS-EB2	46 ~49,XX,+13,+14,+mar1,+mar2[cp6]/ 46,XX[14]	N	P	N	N	Very poor	Very high
18	F/88	MDS-SLD	46,XX[20]	P	N	N	N	Intermediate	Low
21	F/64	MDS-MLD	46,XX,del(5)(q22)[4]/46,XX[16]	P	P	N	N	Good	Low
22	F/63	MDS-MLD	46,XX[20]	P	P	N	N	Good	Low
29	M/67	MDS-SLD	46,XY,del(20)(q11.2)[19]/46,XY[1]	N	N	N	N	Good	Low
31	F/84	MDS-SLD	46,XX[20]	P	N	N	N	Intermediate	Intermediate
32	F/56	MDS-EB-2	46,XX[20]	N	N	N	P	Intermediate	Very high
35	M/66	MDS-U	46,XY[20]	P	N	N	N	Intermediate	Low
37	M/85	Transformation to AML	40 ~ 44,XY,der(5)t(5;?)(q15;?),-7,-12, der(12)t(12;?)(q23;?),-13,-16,-17, der(17)t(17;?)(p13;?),-18,-19,-20,-20, der(21)t(21;?)(q22;?),-22,der(22) t(22;?)(p11.2;?),+mar1,+mar2,+mar3[cp19]/ 46,XY[1]	P	P	P	P	Very poor	Very high
38	F/66	MDS-SLD	46,XX[20]	P	N	N	N	Intermediate	Low
43	F/76	MDS-SLD	46,XX[20]	P	N	N	N	Intermediate	Low
45	M/76	MDS with isolated del(5q)	46,XY[20]	N	P	N	N	Good	Low
48	F/46	Transformation to AML	48,XX,+8,+21[2]/47,XX,+21[18]	N	N	N	N	Intermediate	Very high
50	M/54	MDS-SLD	46,XY[20]	P	N	N	N	Intermediate	Very low

WHO, World Health Organization; MDS-SLD, Myelodysplastic syndrome with single lineage dysplasia; MDS-MLD, Myelodysplastic syndrome with multilineage dysplasia; MDS-RS, Myelodysplastic syndrome with ring sideroblasts; MDS-EB-2, Myelodysplastic syndrome with excess blasts - 2; MDS with isolated del(5q), Myelodysplastic syndrome with isolated del(5q); MDS-U, Myelodysplastic syndrome unclassifiable; Transformation to AML, transformation to acute myeloid leukemia; -5, monosomy 5; del(5q), deletion of long arm of chromosome 5; -7, monosomy 7; del(7q), deletion of long arm of chromosome 7; N, negative FISH signal; P, positive FISH signal; IPSS-R, Revised International Prognostic Scoring System.

**Table 3:** Clinical Features of MDS Patients from CC and FISH

Clinical features	Chromosomal abnormalities by conventional cytogenetic and FISH technique						
	Normal (n=29)	Abnormal (n=21)	*p*	Isolated chromosome 5 abnormalities (n=13)	*p*	chromosome 7 abnormalities (n=3)	*p*
Gender			0.044*		0.041*		0.226
Male, n (%)	20 (69.0)	8 (38.1)		4 (30.8)		1 (33.3)	
Female, n (%)	9 (31.0)	13 (61.9)		9 (69.2)		2 (66.7)	
Age, median (range)	62 (28-93)	66 (46-88)	0.105	66 (54-88)	0.064	70 (56-85)	0.408
Clinical data, median (range)							
Percentage blast of BM	2.0 (0.0-19.0)	1.0 (0.0-90.0)	0.135	0.0 (0.0-2.0)	0.003*	19.0 (3.0-22.0)	0.010*
Hb (g/dL)	8.0 (4.2-12.3)	8.9 (4.6-13.4)	0.088	9.2 (4.6-13.4)	0.019*	7.8 (6.5-8.9)	0.808
WBC count (10^9^/L)	4.4 (1.1-18.2)	4.7 (1.8-209.8)	0.651	4.4 (2.4-6.9)	0.096	6.6 (3.0-47.7)	0.459
Platelet count (10^9^/L)	48.0 (2.0-442.0)	95.0 (17.6-389.0)	0.109	96.0 (17.6-389.0)	0.153	77.0 (44.0-225.0)	0.815
ANC (10^9^/L)	1.8 (0.1-15.1)	2.6 (0.8-30.5)	0.316	2.0 (1.0-5.0)	0.21	3.2 (1.3-30.5)	0.472

*, *p*-value less than 0.05 was considered statistically significant; Percentage blast of BM, percentage blasts of bone marrow; Hb, Hemoglobin; WBC, white blood cells; ANC, absolute neutrophil count.

## Discussion

Chromosomal abnormalities have been studied in adult MDS patients in many countries. Whereas there are few studies reported in the Thai population, especially in the upper northern part of Thailand. According to the previous studies, the chromosomal abnormalities by CC technique were found in 47% in Spanish MDS patients. Among which, the most common abnormalities were +8, del(5q), -7/del(7q), -7, -Y and del(7q) (Solé et al., 2005). Elnahass (2018) found that 46.0% of 50 MDS Egyptian patients had clonal karyotypic abnormalities. The most frequent abnormalities were abnormalities of chromosome 5 (-5/del(5q)) (14.0%) (Elnahass and Youssif, 2018). In Thailand, Intragumtornchai et al., (1998) analyzed bone marrow from 34 MDS patients from 5 hospital centers in Thailand by CC technique and found that 15 patients had abnormal karyotype (44.1%). The most common abnormalities were -7 (26.7%) and +8 (26.7%). However, by CC technique, this study found that the incidence of chromosomal abnormalities was less than the population in previous studies (16.0%). Each abnormality was found in only one case; therefore, the results cannot identify the most common chromosomal abnormalities in this population. The frequency and pattern of chromosomal abnormalities depend on many factors such as environmental factors, genetic background, the number of specimens, and laboratory protocol. Detection of chromosomal abnormalities using CC technique in the large sample size of MDS patients in upper northern Thailand should be study in the future. Chromosomal analysis in a larger population may help to detect specific abnormalities in the MDS patient population. The frequencies of chromosomal abnormalities calculated from the all patients in each population by CC were shown in Figure 2. From this data, the frequency and pattern of chromosomal abnormalities were different in each population (Intragumtornchai et al., 1998; Sendi et al., 2002; Solé et al., 2005; Jung et al., 2011; Rashid et al., 2014; Elnahass and Youssif, 2018). From all of 50 patients in this study, the chromosomal abnormalities were found in 8.0% of the single, 2.0% of the double, and 6.0% of the complex karyotypes by CC. These results agree with many previous studies that the frequencies of single and complex abnormalities were greater than double abnormalities. Complex abnormalities were categorized as a very poor cytogenetic risk group, poor response to treatment, and low survival rate.

Because the CC technique has limitations, consequently, FISH by probes specific for commonly abnormalities regions can help to identify an aberration that has not been seen by the CC technique (Bridge, 2008). Abnormalities of -5/del(5q) and -7/del(7q) are frequently observed in MDS (Delforge, 2003). In this study, detection of chromosomal abnormalities combining CC and FISH techniques would increase the result of abnormal of chromosome from 16.0% to 42.0%. The most of patients with chromosomal abnormalities were MDS-SLD according to WHO 2016 guidelines. When considered in the IPSS-R risk group, most of the patients were intermediate cytogenetic risk and low prognosis risk group.

When compared MDS patients having chromosomal abnormalities with those having normal karyotype in this study, female were greater than male significantly in patient with chromosomal abnormalities (*p* = 0.044). This result agreed with the reported from MDS patients in Spain which found that number of female with chromosomal abnormalities higher than male (Costa et al., 2010), but the result contrasted with MDS patients in India which found chromosomal abnormalities in male were higher than those in female (Vundinti et al., 2009). However, the results from Spain and India did not compare the gender of MDS patients with statistics.

Chromosome 5 is one of the most common chromosomal abnormalities in MDS patients (Hu et al., 2016; Narayanan, 2017; Elnahass and Youssif, 2018). Abnormalities of chromosome 5 are important selecting treatment. This study were interested in clinical characteristics of MDS patients with isolated chromosome 5 abnormalities, therefore MDS patients with other chromosomal abnormalities were excluded from statistical analysis (Table 3). The results showed that MDS patients with isolated chromosome 5 abnormalities had a lower percentage blast of BM (*p* = 0.003) and higher Hb levels (*p* = 0.019) than those with normal karyotype. In this study, the median value of clinical data of MDS patients with isolated chromosome 5 abnormalities show a low percentage blast of BM, elevated platelet count, high Hb levels, and slightly low of WBC count. These features corresponded with MDS patients with isolated del(5q). The clinical features of MDS patients with isolated del(5q) consist of BM blast less than 5%, normal or elevated platelets, mild leukocytopenia, low risk for transformation to AML and commonly favorable risk if not show other chromosomal abnormalities (Haase, 2008). By IPSS-R, low prognosis risk group has a prognostic risk score at 1.5-3.0 (Greenberg et al., 2012). From 13 patients with isolated chromosome 5 abnormalities, 11 of them showed a low risk group. Many previous studies found that the frequency of MDS with del(5q) were more than those isolated -5 (Kantarjian et al., 2009; Galvan et al., 2010; Jung et al., 2011). In contrast to these previous studies, this study found isolated -5 higher than the isolated of del(5q) (47.0% VS 17.6%). However, prognostic risk of MDS patients also depend on other chromosomal abnormalities such as -Y, and del(11q) in the very good cytogenetic risk group or del(12p), and del(20q) in the good cytogenetic risk group. The patients with isolated chromosome 5 abnormalities in this study may have other chromosomal abnormalities which cannot be found by CC. Therefore, investigation on other chromosomal abnormalities by FISH in Thai MDS patients need to be carried out in further studies. Moreover, the prognostic risk also concerning the mutation of gene such as splicing machinery genes or genes involved in epigenetic regulation of transcription (Malcovati et al., 2011; Thol et al., 2012; Martin et al., 2017).

In this study, MDS patients with chromosome 7 abnormalities were found in 3 cases, 1 of isolated del(7q) and 2 of -7 and del(7q) with complex karyotype. Because this study found only 1 patient with isolated chromosome 7 abnormality, therefore, statistical analysis cannot compare the clinical features of this patient with those with normal karyotype. However, when comparing all of chromosome 7 abnormalities patients with normal chromosome patients, the clinical features found a significantly high percentage blast of BM (*p* = 0.010) in patients with chromosome 7 abnormalities (Table 3). Percentage blasts of BM were associated with survival and AML evolution of patients (Greenberg et al., 2012). High percentage blasts showed high risk to progression to AML (Narayanan, 2017). Nevertheless, the significant difference of percentage blast of BM in this study may be due to influence from complex abnormalities. The previous studied found that abnormalities of chromosome 7 were found in part of complex chromosomal abnormalities (Haase et al., 2007; Jung et al., 2011).

The benefit of detecting chromosome 5 and 7 abnormalities is that detection informs the clinician choice of suitable management. Both CC and FISH techniques have benefited by the detection of chromosomal abnormalities. From CC technique, this study detected only one patient with del(5q). While using FISH technique, 3 cases of MDS patients with normal chromosome by CC had del(5q), therefore, these patients were classified as MDS with isolated del(5q). However, the patient who had del(5q) by CC was found -5 by FISH, then this patient was not grouped in MDS with isolated del(5q). From this information, FISH is effective in classify patient in MDS with isolated del(5q). MDS patients with del(5q) are sensitive to lenalidomide treatment (Komrokji et al., 2013). Moreover, FISH can increase abnormalities of chromosome 7 detection. One patient in this study showed normal chromosome by CC but showed del(7q) by FISH. Therefore, the IPSS-R prognostic risk group of this patient was changed from high risk group when detected chromosome by CC to very high-risk group when detected those by FISH. The patients with chromosome 7 abnormalities must be treated with more aggressive treatment to neutralize disease progression (Adema et al., 2013). However, a limitation in this study was relatively small sample size. A larger sample size and a study of molecular genetic technique such as gene mutation might provide more information in the future.

In conclusion, the results of this study showed that the frequency and pattern of chromosomal abnormalities of MDS patients in upper northern Thailand were different from other populations. MDS with isolated chromosome 5 abnormalities had clinical characteristics corresponding with patients in the good prognostic risk group. While, MDS patients with chromosome 7 and complex abnormalities showed high a percentage blast of BM which high risk to progression to AML. This study provides information of MDS patients in the upper northern Thailand for identifying prognosis and choosing the proper treatment for patient in this population group. Combined CC and FISH techniques detected chromosomal abnormalities with greater frequency than when either technique is used alone.
